# Using ultra-low frequency waves and their characteristics to diagnose key physics of substorm onset

**DOI:** 10.1186/s40562-017-0089-0

**Published:** 2017-10-24

**Authors:** I. J. Rae, K. R. Murphy, Clare E. J. Watt, Ian R. Mann, Zhonghua Yao, Nadine M. E. Kalmoni, Colin Forsyth, David K. Milling

**Affiliations:** 1 Dept. of Space and Climate Physics, Mullard Space Science Laboratory, University College London, Holmbury St. Mary, Dorking, Surrey, RH5 6NT UK; 2 Goddard Space Flight Center, NASA, Greenbelt, USA; 3 Department of Meteorology, University of Reading, Reading, UK; 4 Department of Physics, University of Alberta, Edmonton, Canada; 5 Space Science, Technologies and Astrophysics Research (STAR) Institute, Université de Liège, Liège, Belgium

## Abstract

Substorm onset is marked in the ionosphere by the sudden brightening of an existing auroral arc or the creation of a new auroral arc. Also present is the formation of auroral beads, proposed to play a key role in the detonation of the substorm, as well as the development of the large-scale substorm current wedge (SCW), invoked to carry the current diversion. Both these phenomena, auroral beads and the SCW, have been intimately related to ultra-low frequency (ULF) waves of specific frequencies as observed by ground-based magnetometers. We present a case study of the absolute and relative timing of Pi1 and Pi2 ULF wave bands with regard to a small substorm expansion phase onset. We find that there is both a location and frequency dependence for the onset of ULF waves. A clear epicentre is observed in specific wave frequencies concurrent with the brightening of the substorm onset arc and the presence of “auroral beads”. At higher and lower wave frequencies, different epicentre patterns are revealed, which we conclude demonstrate different characteristics of the onset process; at higher frequencies, this epicentre may demonstrate phase mixing, and at intermediate and lower frequencies these epicentres are characteristic of auroral beads and cold plasma approximation of the “Tamao travel time” from near-earth neutral line reconnection and formation of the SCW.

## Background

Substorm expansion phase onset is traditionally identified in ionospheric optical observations by the brightening of a pre-existing auroral arc or the formation of a new arc (Akasofu 1964, 1977). This brightening is accompanied by ultra-low frequency (ULF) waves in the Pi2 band (40–150 s period; Jacobs et al. 1964) that develop when the cross-tail current is diverted towards the ionosphere during the dipolarisation of the nightside magnetospheric field and formation of the substorm current wedge (SCW; e.g. Atkinson 1967; McPherron et al. 1973). Following substorm onset, these Pi2s have also been shown to couple to the plasmasphere forming a plasmaspheric cavity mode (Yeoman and Orr 1989) and have been linked to plasma sheet flows in the tail (Kepko and Kivelson 1999; Murphy et al. 2011b), presumably corresponding to transient magnetotail reconnection (Keiling et al. 2006). At substorm onset, the general characteristics of Pi2s are used to determine the large-scale characteristics of the SCW, including the locations of the upward and downward field-aligned current (FAC) elements and the central meridian (e.g. Lester et al. 1983, 1984). However, the precise relationship between Pi2 waves and the first observable changes of auroral arc brightening is limited by a number of observational constraints. Pi2 waves have periods of ~ 40–150 s, which are of the order of the “2-min problem” (Ohtani 2004) surrounding onset. Additionally, the integrated ionospheric currents that magnetometers measure are inherently noisy and so it is challenging to identify the “first” evidence of ULF wave activity when the low-amplitude signals are growing out of a noisy or elevated background. Linking Pi2 to substorm onset is further complicated by the plethora of physical phenomena that have been suggested to produce ULF pulsations in the ionosphere. Shorter period ULF wave bands such as the Pi1B (~ 1–10 s) period band, part of the overall Pi1 band (1–40 s), offer a means to reduce the onset timing uncertainty (e.g. Bösinger 1989; Lessard et al. 2006; Posch et al. 2007) in a localised region close to onset (e.g. Bösinger and Yahnin 1987; Arnoldy et al. 1987) and more importantly are not directly linked to other magnetospheric phenomena.

The long-period part of Pi1/short-period part of Pi2 ULF wave band (hereafter referred to as the long-period Pi1 band or Pi1-2 for brevity) was first investigated by Milling et al. (2008). In this paper, a discrete wavelet transform (DWT) based upon the Pi2 algorithm outlined by Nose et al. (1998) was used to investigate the entire ULF wave spectrum during substorm expansion phase onset. The Meyer wavelet was used for the analysis as it closely resembles the impulsive nature of nightside ULF waves and has excellent timing resolution, and hence is ideal for defining any “onset”. Milling et al. (2008) found that the first ULF wave band to rise above a pre-determined noise threshold was in the long-period Pi1 wavelet band, and when the analysis was extended across all available magnetometers, the long-period Pi1 waves had a clear and coherent onset that spread out from an epicentre in the auroral zone.

Murphy et al. (2009a) presented an automated algorithm based on the Meyer wavelet (christened the automated wavelet estimation of substorm onset and magnetic events—AWESOME) and used it to compare the onset timing and location of long-period Pi1 waves with global auroral intensification times detailed in the Frey substorm listings (Frey et al. 2004; Frey and Mende 2006). Murphy et al. found that the epicentre of ULF wave activity was co-located with the initial location of global auroral intensification observed at substorm expansion phase onset, as determined by the global auroral imaging from the IMAGE-FUV instrument. However, the onset of long-period Pi1 ULF wave activity generally occurred ~ minutes prior to the global auroral intensification during substorm expansion phase onset. Liou and Zhang (2009) argued that the temporal resolution of the IMAGE-FUV instrument meant that the ULF wave onset and substorm onset could occur simultaneously. However, Murphy et al. (2009b) demonstrated that even with a more in-depth consideration of the temporal cadence of each instrument in the study, in general ULF wave onset always precedes global auroral breakup.

Rae et al. (2009a) studied the relationship between smaller scale auroral features observed minutes prior to auroral breakup and the onset of Pi1-2 ULF waves observed by both Milling et al. (2008) and Murphy et al. (2009a, b). After identifying the onset arc, Akasofu (1964) and Rae et al. (2009a, b) showed that the onset of Pi1-2 ULF waves occurred at the same time and in the same location as “auroral beads” (Henderson 1994) which developed azimuthally along the onset arc. Rae et al. (2009b) and Walsh et al. (2010) went on to demonstrate the rapid connectivity of the equatorial magnetosphere to the ionosphere during substorm onset, showing that the Pi1-2 ULF onset observed on the ground and at the conjugate point in the equatorial magnetosphere occurred within the timing uncertainty defined by the AWESOME algorithm. Further studies of the onset of the ULF waves shows that the exponential growth in ULF wave amplitudes occurs immediately prior to the onset of an auroral substorm (Rae et al. 2011). Finally, it has been shown that both the ULF wave amplitudes and auroral brightness grow exponentially at the location of ionospheric substorm onset (Rae et al. 2012; Kalmoni et al. 2015, 2017). Although the relationship between the enhancement of ULF waves and auroral brightness is no longer in doubt (see review by Rae and Watt 2016), the relative timing and localisation of Pi1, Pi1-2 and Pi2 waves across the auroral zone at substorm onset have not yet been investigated.

The arrival time of Pi2 ULF waves has been used by Chi et al. (2009) to infer a magnetospheric source of substorm onset in the magnetotail by investigating the latitudinal dependence of the first peak in Pi2 amplitude observed by ground magnetometers along a meridian. In their paper, Chi et al. (2009) found that there is a strong latitudinal dependence of this first Pi2 peak with stations in the auroral zone observing this peak ~ 1 min prior to both higher and lower latitudinal stations. By considering the travel path preserving the most wave energy using the Tamao travel time (e.g. Tamao 1964), these authors concluded that the distances and timing that were most consistent with the latitudinal profile of the first Pi2 peak were ~ 1–3 min prior at 15–25 RE distances in the magnetotail. This technique is often referred to as “magnetoseismology”, and for a comprehensive review of this, we direct the reader to Menk and Waters (2013). Here, we study not the first notable peak in wave amplitude, but the onset of wave activity as defined by the AWESOME algorithm (Murphy et al. 2009a), and we extend the analysis from Pi1 waves, through the long-period Pi1 band, to Pi2 waves to study all frequencies (see Murphy et al. 2011a).

Hence, as discussed above, there have been two specific drivers of two specific wave bands discussed in the literature; Pi1/2 waves are in general associated with the substorm onset arc that maps in the near-Earth region between stretched and dipolar fields where current disruption plays a key role (e.g. Roux et al. 1991), whereas Pi2 waves are related to near-Earth neutral line physics driven by magnetotail reconnection (e.g. Hones 1976). Determining differences between these two ULF wave bands therefore potentially represents a novel method to distinguish between these two substorm onset mechanisms.

To understand all aspects of ULF wave studies, one must study the timing, latitudinal, longitudinal and frequency-dependent onset characteristics of ULF waves and, to date, no such measurement of all four quantities has been made. In this paper, we study the frequency-dependent onset of ULF waves as a function of time and location. Specifically, we present magnetic observations of a small substorm on 5 March 2008, where we utilise AWESOME to define the 2D ionospheric projection of the first rapid increase in magnetic oscillation amplitude in the Pi1, Pi1-2 and Pi2 ULF wave bands close to the auroral onset. Using this technique, we directly compare both the absolute and relative timing and location characteristics of all three essential ULF wave bands during substorm expansion phase onset. We find that the Pi1-2 ULF wave band occurs first at auroral latitudes, but Pi2 ULF waves may be observed concurrently at lower latitudes, to within the timing uncertainty of the technique. We postulate that Pi1-2 ULF waves are critically important for defining the timing and location of the auroral substorm expansion phase, but that the low-latitude Pi2 onset may provide valuable information on the location of the plasmapause. We discuss these results in terms of previous findings.

## Instrumentation and large-scale morphology

**Fig. 1:**
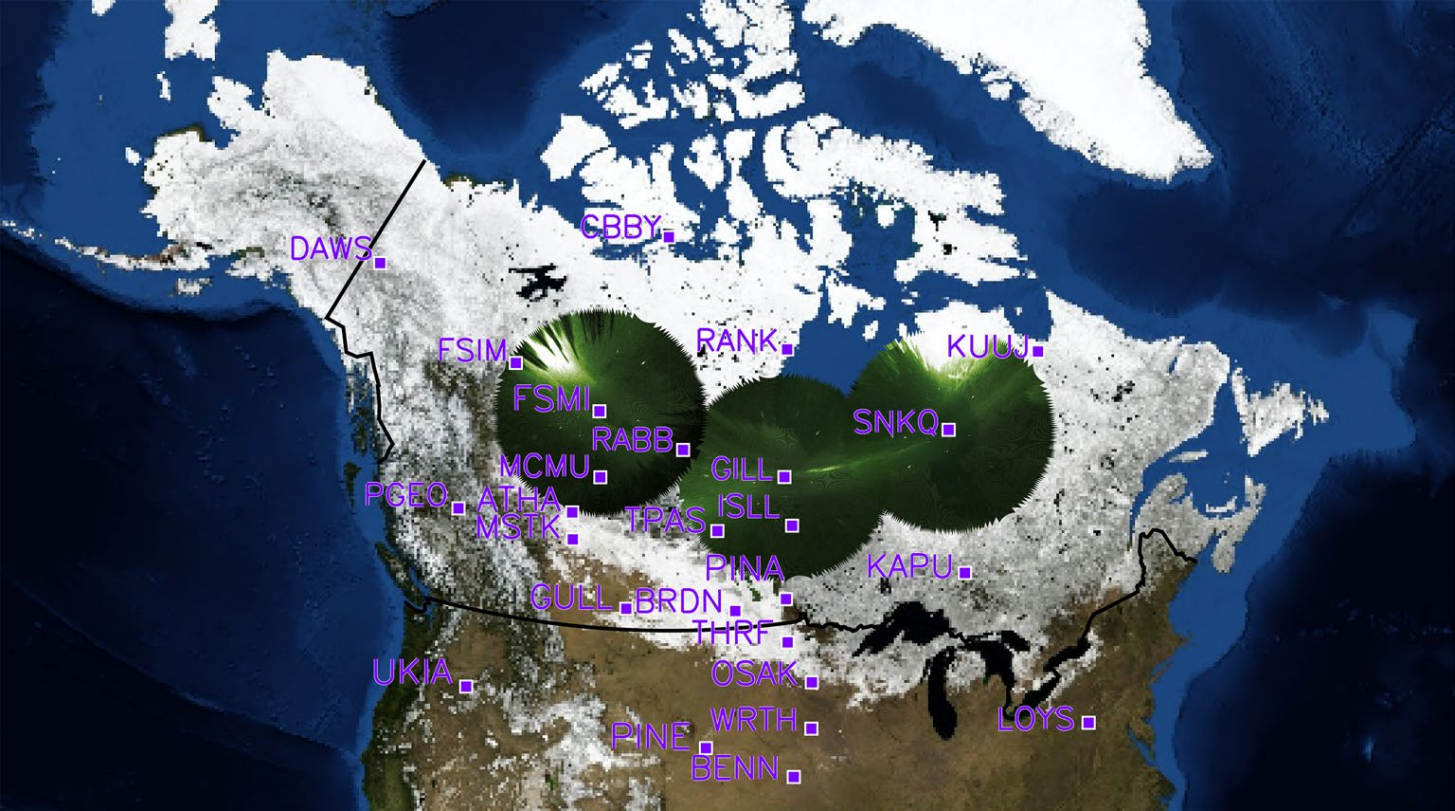
Locations of the ground magnetometers from the CARISMA, THEMIS, CANMOS and McMAC magnetometer chains (for details see text). Overplotted in the figure are data from the GILL THEMIS ASI at 06:04:20 UT which approximately correspond to the time of auroral brightening in this substorm event

**Fig. 2:**
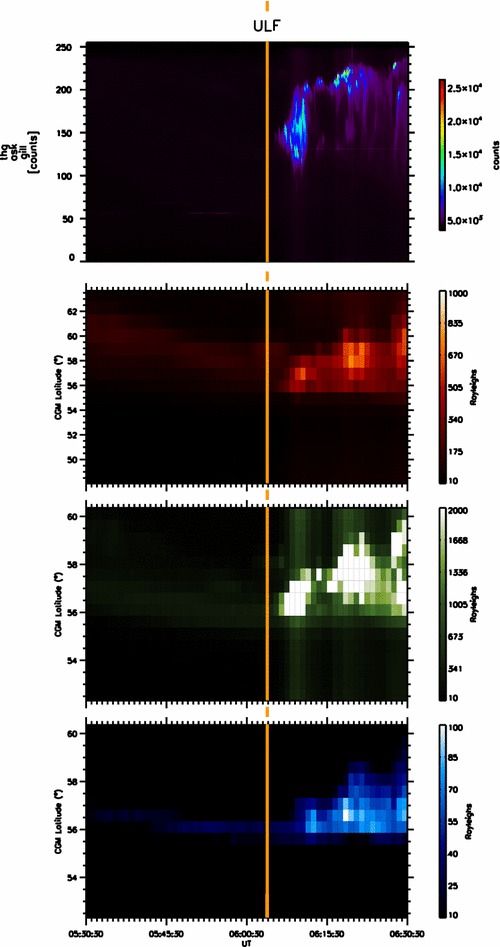
North–south slices (keograms) from the GILL THEMIS ASI and GILL NORSTAR MSP from 05:30:30 to 06:30:30 UT. From top to bottom, the figure shows data from the white-light GILL ASI, together with the 6300 A (red), 5577 A (green) and 4861 A (blue) emission lines from the GILL NORSTAR MSP. The orange vertical line denotes the first observation of ULF waves during this interval, at 06:04:20 UT, discussed later in the text

In this study, we use magnetometer stations from the Canadian array for real-time investigation of magnetic activity; CARISMA—Mann et al. 2008), time history of events and macroscale interactions during substorms (THEMIS; Angelopoulos 2008; Sibeck and Angelopoulos 2008), GMAG (Russell et al. 2008) and EPO (Peticolas et al. 2008) magnetometer chains, together with the Mid-continent Magnetoseismic Chain (McMAC) and stations in the Canadian magnetic observatory system (CANMOS) magnetometer arrays. Figure 1 shows the locations of the ground magnetometers used in this study for context, together with their four-letter station IDs. Overplotted on this map is a white-light auroral image from the Gillam THEMIS All-Sky Imager (Mende et al. 2008) at the approximate time of initial auroral brightening, indicating that the brightening of the onset arc in this case study is slightly to the east of the Gillam ASI meridian. Structuring of the brightening auroral arc subsequently develops into latitudinally confined periodic azimuthal undulations, termed “auroral beads” after Henderson (1994), and subsequently re-discovered in the THEMIS era (e.g. Rae et al. 2009a, b, 2010, 2011 Murphy et al. 2015; Kalmoni et al. 2015, 2017). Following the development of these auroral beads, auroral breakup occurs. The auroral surge forms and expands poleward as expected during the substorm expansion phase. The faint auroral arc located approximately 2° poleward of this initiation region and conjugate to the FCHU station remains quiet on the same timescale, indicating that this substorm expansion phase onset does not involve any activation of the poleward boundary of the auroral oval (e.g. Nishimura et al. 2010). Figure 2 shows a summary of the auroral data showing the onset of the auroral substorm. For additional supporting information, we refer the reader to Fig. 1 of Rae et al. (2012). Figure 2 shows a series of north–south slices (keogram) through zenith in the Gillam ASI, as well as the keograms taken by the Gillam NORSTAR meridian scanning photometer in the 6300, 5577 and 4861 A wavelengths (Donovan et al. 2003). Figure 2 shows the intensification and subsequent poleward expansion of the auroral surge ~ 0604–0605 UT, which occurs following the contemporaneous long-period Pi1/short-period Pi2 ULF wave onset that marks the initiation of the substorm (e.g. Milling et al. 2008) and which is discussed below.

## AWESOME magnetic timing

The discrete wavelet transform (DWT) utilised by AWESOME uses a Meyer basis wavelet to decompose a non-stationary signal into discrete wavelet coefficients that are localised in time and band limited in frequency in order to objectively define the first time ULF wave amplitudes rise above a noise threshold, which we define as the onset of ULF waves, during magnetic substorms (see Murphy et al. 2009a for details). For each individual magnetometer station, the quietest interval preceding the event during the UT day is selected (i.e. an interval that does not include significant ULF wave variations) and the power in each wavelet period band is calculated during that interval. A threshold is then defined independently for each wavelet band as the mean plus two standard deviations of the power. Onset in a specific ULF wave band is then defined as the initial time at which the wavelet power coefficient rises above the quite time threshold. Our definition ensures a 98% confidence level that the signal which rises above this threshold represents a statistically significant signal above quiet time fluctuations. A discrete onset time is defined as the centre of the interval when the wavelet coefficient first exceeds this threshold, and the uncertainty is defined as plus or minus the temporal width of the coefficient band. Statistically, the ULF power spectra around onset are characteristic of a power law; neither the Pi1, Pi1-2 or Pi2 frequency bands are preferred at substorm onset nor is there any clear break in the ULF wave spectra (Murphy et al. 2011b). The AWESOME algorithm is designed so that the entire ULF wave spectrum is analysed without identifying an a priori ULF wave band for study during any particular event. In this paper, the AWESOME algorithm is utilised to detail the absolute and relative timing in the 12–48 s, 24–96 s and 48–192 s wavelet bands, timing uncertainties for each of the three wavelet bands being ± 8 s, ± 16 s and ± 32 s, respectively.

## A case study in relative ULF wave timing

**Fig. 3:**
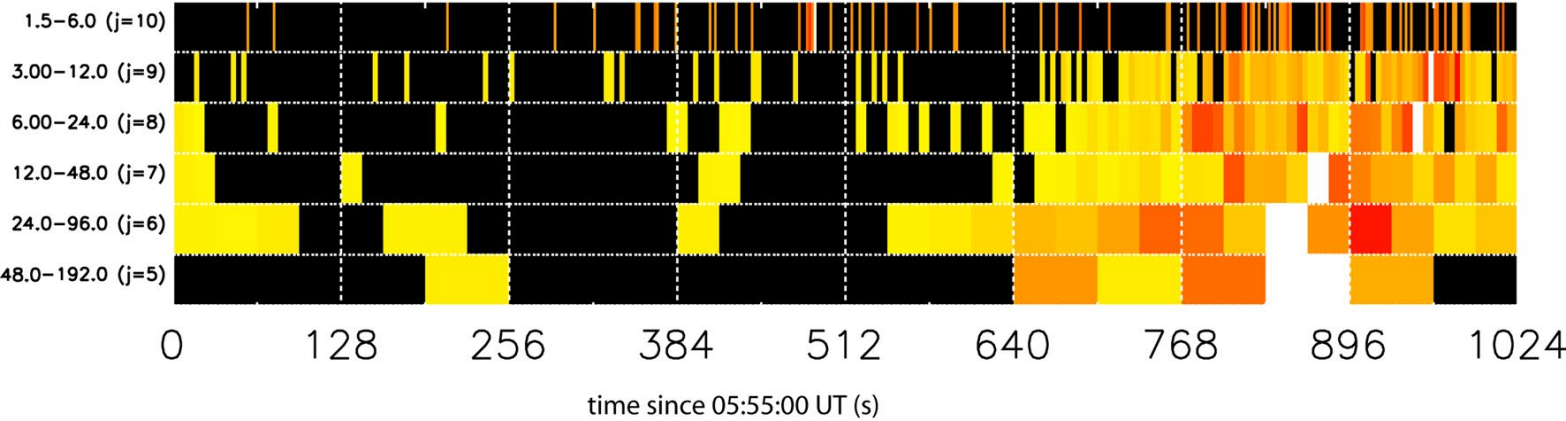
AWESOME (Murphy et al. 2009a) analysis of the substorm in 2008-03-05/06:04:20 UT, run from 05:55:00 UT. Displayed in the figure are wavelet-derived power spectra as a function of time and wavelet period (s) or j (wavelet basis). The colours represent normalised wavelet coefficients, whereby black represents wavelet coefficients below the pre-determined threshold for each j and yellow-orange-red-white are coefficients rising above the threshold in increasing amplitude

Figure 3 shows exemplar AWESOME power spectra for the GILL magnetometer for six ULF wave bands spanning the Pi1–Pi2 ULF wave periods and starting at 0555 UT. As with all magnetometer time series, some fluctuating noise exists in all ULF wave bands that precede ULF wave onset, most notably in the 6–24 s period band. However, no signal rises above the pre-determined noise threshold for more than a couple of intervals until the 24–96 s ULF wave signal at 544 ± 16 s. This onset is followed by the 48–192 s signal at 640 ± 32 s, and finally by the Pi1 onset at 656 ± 8 s. Note that the onset of higher frequency bands is recorded at the GILL station at 648 ± 4 s in the 6–24 s ULF wave band, and later in the 3–12 s ULF wave band, but signals in these bands are not observed across the whole magnetometer network and are only observed locally to the onset location shown in Fig. 1, as found by Bösinger and Yahnin (1987) and Arnoldy et al. (1987). In summary, it can be observed from Fig. 3 that ULF waves in the Pi1-2 band occur first at the GILL magnetometer, followed ~ 100 s later by the onset of waves in the traditional Pi2 and Pi1 ULF wave bands.

**Fig. 4:**
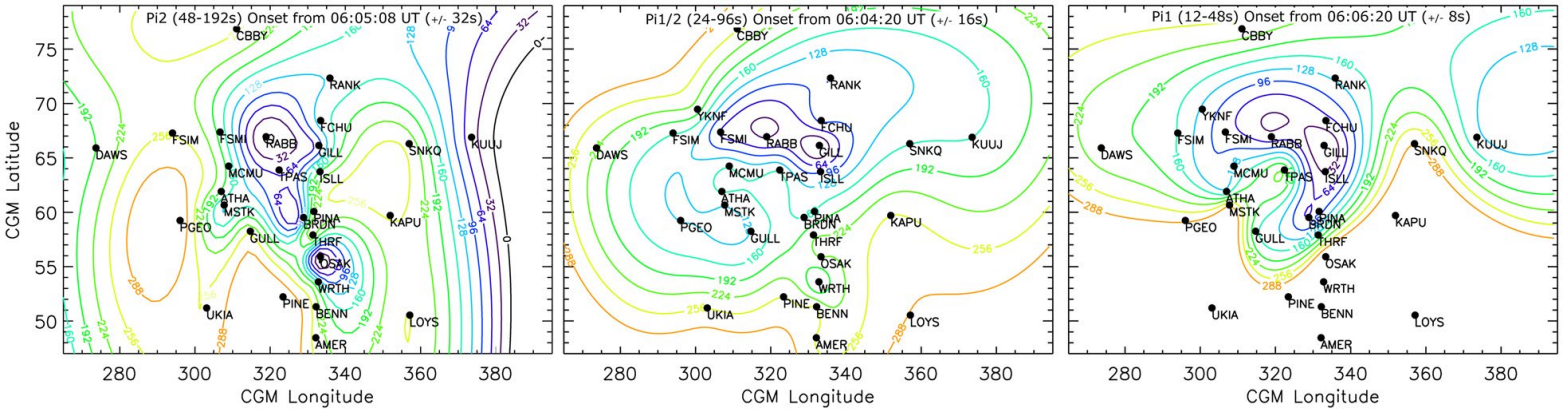
A minimum curvature fit for the onset times in the (left) *j* = 5 or 48–192 s, (centre) *j* = 6 or 24–96 s, and (right) *j* = 7 or 12–48 s period bands as derived at each magnetometer station. Coloured, annotated contours represent the onset times, and the contours of each panel are separated by 32 s to show consistent onset contours across wave bands. The maximum error in this fit is of the order of seconds and hence represents the actual onset time at each station within stated errors. Stations that did not record an onset in the right-hand panel at lower latitudes are not used in this analysis

By analysing the onset of each wavelet band at each station, we estimate the onset time of ULF wave activity in each wavelet band across the entire combined magnetometer array. A minimum curvature fit is a general term that refers to an interpolation in space between an irregularly spaced set of points that minimises the collective distance between the measurement points, whilst still passing through each measurement point. We use this to describe the shape of the “epicentre” from our irregularly spaced ground magnetometer measurement points. We use the IDL procedure min_curv_surf, which interpolates an irregularly gridded set of points (such as those obtained from ground magnetometers) over a spherical surface in our case, the Earth’s surface. In this way, a visual representation of the onset times can be made, whilst still preserving the measured onset times at each and every magnetometer station. We refer the reader to Milling et al. (2008) for a detailed explanation of this fitting technique and their robust results. Figure 4 shows this minimum curvature fit to the onset times in the 48-192 s, 24–96 s and 12–48 s period bands for all available magnetometer stations, left, middle and right, respectively. Each contour is 32 s apart to make comparisons easier, but the contours are calculated from the first observation of each period band separately as indicated in the figure caption.

Figure 4a shows the minimum curvature fit to the Pi2 arrival times, relative to the first Pi2 arrival time at RABB at 06:05:08 UT ± 32 s, whereas Fig. 4b shows timed relative to the Pi1-2 arrival time at GILL-RABB at 06:04:20 ± 16 s, and Fig. 4c shows timed relative to the Pi1 arrival time at GILL at 06:06:20 ± 8 s. There are notable similarities and differences between the onset of ULF waves in each of the three Pi1 and Pi2 wave bands, but for each wave band at least one location of the epicentre is well aligned with the onset arc as shown in Fig. 1. In the case of the Pi1/2 wave band, the epicentre is indeed well aligned with the east–west onset arc. We note here that the closed contours at RABB and GILL are part of the same epicentre, as each magnetometer observes the onset signature at the same time. Hence, these closed contours are simply a result of the minimum curvature fit to the data, rather than any physical process. This alignment of the Pi1/2 epicentre is consistent with the results of Murphy et al. (2009a, b), Rae et al. (2009a, b, 2010, 2011) and Walsh et al. (2010), amongst others, that the long-period Pi1/short-period Pi2 waves are initially seen in the ionosphere at the location of substorm onset. Interestingly, however, the times at which these epicentres are initiated are significantly different. Using AWESOME, we can account for the uncertainty in timing, and we demonstrate that in this case study the Pi1-2 wave band is observed ~ 1 min prior to the Pi2 wave band and ~ 2 min prior to the Pi1 wave band. Rae et al. (2009a, b) showed that the onset of Pi1-2 wave powers was commensurate with the development of auroral beads and postulated that the onset of Pi2 waves was associated with auroral breakup. Again, this is consistent with the results presented in this paper.

However, the Pi1, Pi1-2 and Pi2 arrival patterns are quite dissimilar across a wide range of latitudes and longitudes. Indeed, there is a second Pi2 epicentre observed at low latitudes centred around OSAK, where Pi2 onset is observed at the same time as the auroral Pi2 onset location at RABB. The onsets of Pi2 waves at stations between OSAK and RABB (THRF and BRDN) are later; there are sufficient data in this part of the plot that we may have confidence in the contours as drawn. We discuss the implications of this Pi2 onset distribution in the “Discussion” section.

The patterns of onset of the Pi1 waves shown in Fig. 4c differ from both the Pi1-2 and Pi2 ULF wave onsets. Firstly, the AWESOME-determined Pi1 onset does not extend to the lower latitudes that the Pi1-2 and Pi2 onsets did in this case study; the lowest latitude station that observed Pi1 onset was PINE at ~ 52° latitude. Secondly, although the high-latitude Pi1 onset exhibits largely the same spatial onset pattern as the Pi1-2 wave onset, both occurring first at GILL and expanding clearly and coherently away from this epicentre region, there is a delay of ~ 2 min after the Pi1-2 onset.

In summary, it can be observed from Fig. 4 that Pi1-2 waves are initially confined along the substorm onset arc that is azimuthally aligned with the GILL-RABB-FSMI magnetometers, and the onset signature propagates relatively coherently across the rest of the magnetometer array, which is in keeping with the results shown previously by Murphy et al. (2009a), Rae et al. (2009a, b) and Walsh et al. (2010). Pi2 waves occur later in the substorm and their onset signals propagate significantly faster but still coherently across the array from two epicentres, one centred on the location of the substorm onset arc and another at lower latitudes. Finally, Pi1 waves occur even later in the substorm, are confined near onset and propagate relatively slowly compared to other ULF wave signals.

## Importance of relative timing of wave onsets in substorm physics

In “A case study in relative ULF wave timing” section, we demonstrate that each ULF wave band has significantly different onset times as a function of radial distance away from the auroral onset region. As in numerous previous studies, the epicentre of Pi1-2 ULF wave onset in this case study occurs contemporaneously with the growing auroral bead signature that designates auroral substorm onset, in this case study near the GILL station. These auroral beads, which mark the onset of the substorm in the ionosphere (e.g. Rae et al. 2010; Murphy et al. 2015; Kalmoni et al. 2015, 2017), have been shown to grow exponentially in brightness at the same time as the onset of Pi1-2 ULF waves (~ 0.05 s^−1^; Kalmoni et al. 2015, demonstrating that auroral onset and ULF wave activity are inextricably linked through whichever process(es) cause substorm onset, e.g. Murphy et al. (2009a, b, 2015) and Rae et al. (2009a, b, 2010, 2011, 2012).

This Pi1-2 onset occurs first, but is closely followed by the onset of Pi2 ULF wave activity at two separated epicentres close to the RABB-GILL station and at mid-latitudes at OSAK. The Pi2 waves are observed around 1 min later at the onset station at GILL. Finally, the shorter period Pi1 waves are observed in a region around the GILL-RABB stations.

The primary aim of developing the AWESOME technique was to analyse the entire ULF wave spectra and provide an estimate of uncertainty in the onset time, in addition to setting an objective and quantitative onset criteria. We note here that there is no overlap in uncertainty between the onsets of the different ULF wave bands, demonstrating that the onset of Pi1-2 waves occurs prior to Pi2 waves, and both occur prior to the higher frequency Pi1 wave band consistent with previous studies at single stations (Murphy et al. 2009a, b; Rae et al. 2009a, b; Walsh et al. 2010). We conclude that the Pi1-2 waves are linked to the formation and evolution of auroral beads and are therefore onset driven (e.g. Rae et al. 2009a, b), whereas the longer period Pi2 waves may be related to the evolution of the large-scale current system that develops following onset, most likely the substorm current wedge (e.g. McPherron et al. 1973; Rae et al. 2007; Murphy et al. 2013). The timing difference between auroral beads and auroral breakup during which the SCW begins to form is similar to timing difference between the onset of the Pi1-2 and Pi2 wave bands; beads and Pi1/2 waves occurring contemporaneously and auroral breakup and Pi2 waves occurring 2–3 min later. This further supports our conclusion that Pi1-2 waves are linked to the auroral beads and Pi2 waves are linked to the SCW. Presumably in this scenario, Pi1 waves correspond to the later structuring and filamentation of the current systems that develop into auroral breakup (e.g. Arnoldy et al. 1987; Bösinger 1989).

We further analyse the onset times of these three ULF wave bands in order to highlight the precise relative timing between the onset times as a function of latitude and longitude and reveal additional information on the formation and evolution of the substorm.

**Fig. 5:**
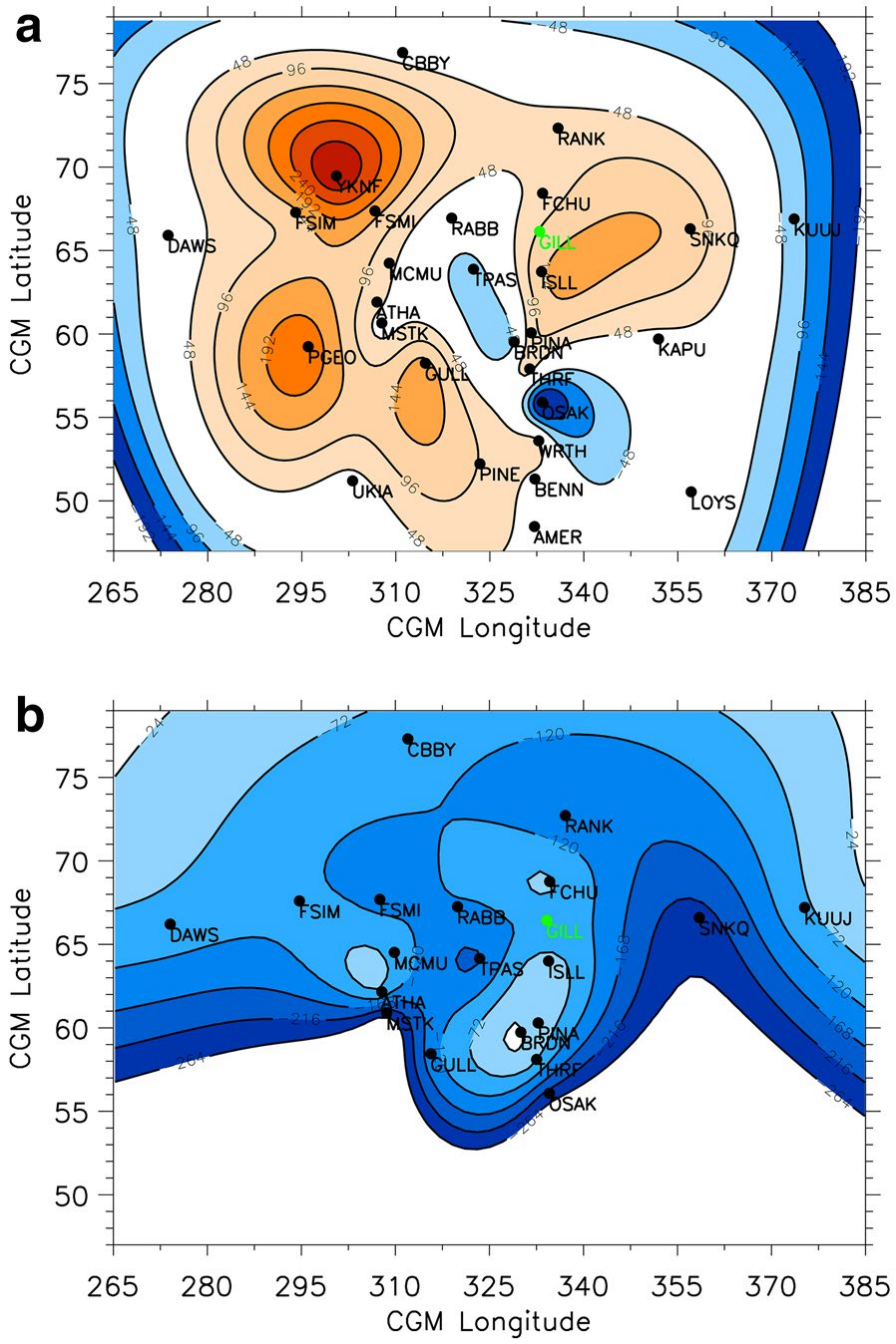
A minimum curvature fit to the difference in onset times across the entire Canadian sector for (**a**) Pi2 and Pi1-2, where red (blue) represents Pi2 waves occurring first (second), and (**b**) Pi1 and Pi1-2, where blue represents Pi1 waves occurring second, with respect to Pi1-2 waves. The uncertainty in the relative timing between ULF wave bands is defined as the summation of each individual ULF wave band uncertainty. Hence, the timing uncertainties associated with each comparison are as follows: Pi1–Pi1-2 is ± 24 s, Pi1-2–Pi2 is ± 48 s. Any regions in white represent locations where the onset of ULF waves in each band is at the same time within the inherent uncertainties outlined here. Each relative timing analysis thus uses contours which start at the first resolvable timing difference outside these different uncertainties, and those that cannot be resolved are marked in white

Figure 5 shows the relative timing between the onset of Pi1-2 waves and their (left) Pi2 and (right) Pi1 ULF wave band counterparts at each magnetometer station. Again, Fig. 5 shows a minimum curvature fit to the relative onset times between each period band. The varying shades of red in Fig. 5a indicate the regions where Pi1-2 onset time is earlier than Pi2 onset time, and the blue represent regions where the Pi1-2 onset time is later than the Pi2 onset times. In Fig. 5b, blue denotes the locations where the Pi1-2 onset is before Pi1 onset, which it is across the entire observable region. Any regions in white are where onset times are the same within the uncertainty and the auroral onset station (GILL) is marked in green to aid the eye since this station first observes both the Pi1-2 onset and the auroral onset at 06:04:20 UT. For further details, we refer the reader to the figure caption.

A number of features stand out in Fig. 5a, most notably that at the auroral and ULF wave onset station Pi1-2 ULF waves clearly lead Pi2 waves by ~ 1 min and across the majority of latitudes and longitudes the Pi1-2 onset leads the Pi2 onset by ~ 1–2 min. However, there appears to be a channel in the ionosphere where Pi2 ULF waves are observed prior to Pi1-2 waves, ranging from TPAS to OSAK and to the west of substorm onset, marked by the blue timing contours in Fig. 5a between 320–345° longitudes.

Figure 5b shows the relative timing between the Pi1-2 and Pi1 ULF wave bands. Figure 5b clearly demonstrates that the onset of Pi1-2 ULF waves occur ~ 1–4 min prior to the higher frequency Pi1 ULF waves, other than at one station (BRDN) which lies within the timing uncertainty contour. Note that the relative timing cannot be calculated below 55° latitude since the Pi1 onsets were only observed over a limited spatial region. The onset of Pi1 ULF waves is delayed longest with respect to the onset of Pi1-2 waves at these lowest observable latitudes.

## Discussion

We present a case study of the relative timing of ULF wave onset across the Pi1 and Pi2 ULF wave bands. These waves are known to play a pivotal role in substorm studies as a diagnostic (Rae et al. 2009a, b, 2010) and a timing mechanism for substorm onset (Milling et al. 2008; Murphy et al. 2009a, b). We find that there are clear epicentres observed in each wave frequency band, and we discuss the ramifications on the generation mechanisms of each of these specific ULF wave bands.

Historically, Pi2 waves have been invoked to carry the current of the substorm current wedge (e.g. McPherron et al. 1973; Rae et al. 2007), related to the onset of NENL reconnection (e.g. Liou et al. 2000; Uozumi et al. 2000, 2004, 2009; Chi et al. 2009). Using magnetoseismology or “Tamao travel times” in a cold plasma, Chi et al. (2009) determined that, by studying a visually determined Pi2 onset time in the ionosphere, constraints on the location of NENL reconnection could be determined. Interestingly, Chi et al. (2009) determined that there were two paths which minimised the time-of-flight of a Pi2: one along the PSBL and one at lower latitudes outside the plasmapause where Alfven speeds are higher than the surrounding plasma sheet. This would lead to the onset of Pi2 waves at two discrete epicentres, in precisely the same configuration as shown in Fig. 4(left). Pi2 waves also appear to be global, being observed throughout the nightside ionosphere, which is consistent with the historical literature suggesting that Pi2 waves are a “global mode” (see Keiling and Takahashi 2011 for a comprehensive review). In this scenario, Pi2 waves at higher latitudes would correspond to the open-closed separatrix shown in Fig. 2b to be situated around 70° latitude (e.g. Blanchard et al. 1995). As shown in Fig. 4(left), this location is not populated with a ground magnetometer, but the closest high-latitude magnetometer station (RABB at 67°) is the first magnetometer to observe the first Pi2 signals, which supports this hypothesis. Moreover, depending upon how stretched the magnetic field is and where NENL reconnection is initiated, the onset of Pi2 waves would potentially be simultaneous or later for the magnetometer station closest to the plasmapause (Chi and Russell 2005; Chi et al. 2009). As travel time magnetoseismology is a strong function of plasma sheet temperature, field line stretching and assumed NENL reconnection location, modelling wave propagation times is beyond the scope of this paper. However, using the results from the literature described above, we suggest that the two epicentres of Pi2 wave activity discovered in this case study, coupled with a meridional channel of early Pi2 arrival times, support the hypothesis of NENL reconnection occurring outside of 15RE in the magnetotail (Fig. 2 of Chi et al. 2009). In this scenario, Pi2 waves are observed either in two epicentres described above, or relatively simultaneously in a single meridian (see Figs. 4a, 5a).

On the other hand, Pi1-2 waves have been exclusively linked to the formation and structuring of the substorm onset arc (e.g. Rae et al. 2009a, b). Specifically, Pi1-2 waves have been linked to the formation of what is termed “auroral beads” after Henderson (1994). These auroral fluctuations have the same temporal periodicity as Pi1-2 ULF waves and initially form azimuthally along the onset arc (Rae et al. 2012). Evident from Fig. 4b is the fact that the Pi1-2 onset occurs initially at GILL, RABB and FSMI stations conjugate to the onset arc. This supports the hypothesis that Pi1-2 waves are the magnetic counterparts of auroral beads. Detailed analysis of these auroral beads (Rae et al. 2010; Kalmoni et al. 2015, 2017) and auroral and plasma sheet dynamics at substorm onset (Rae et al. 2009a; Murphy et al. 2015) has provided strong evidence that auroral beads are the ionospheric manifestation of a plasma instability driven by substorm onset, whether it be in the near-Earth magnetotail (e.g. Lui 1991) or closer to the ionosphere (e.g. Motoba et al. 2012). Since both Pi1-2 ULF waves (e.g. Murphy et al. 2011b; Rae et al. 2011) and auroral beads (Kalmoni et al. 2017) are ubiquitous to substorm onset, the Pi1-2 waves appear to be exclusively related to the substorm onset instability at the inner edge of the plasma sheet (e.g. Samson et al. 1992).

Finally, Pi1 waves occur in minutes following substorm onset during the substorm expansion phase and auroral breakup as the aurora expands poleward and toward the dawn and dusk of the onset region. In a statistical study of 11 years of DMSP electron observations, Wing et al. (2013) showed that following substorm onset there is a sharp increase in wave-driven aurora characterised by broadband electron precipitation (c.f., Newell et al. 2010). Our observations together with those from Wing et al. (2013) indicate that Pi1 waves are related to the filamentary currents generated following substorm onset and localised poleward of the onset arc region (e.g. Arnoldy et al. 1987; Bösinger 1989).

That two separate mechanisms are invoked during substorm onset is not a new concept (e.g. Lui 1991; Pu et al. 1999). Studies often appeal to either one mechanism occurring before the other, such as reconnection-driven current disruption (e.g. Angelopoulos 2008) or via current disruption leading to reconnection subsequently (e.g. Lui 2009). Indeed, these could be independent regions being activated independently (e.g. Murphy et al. 2015). Our results are consistent with the literature that Pi2 waves are driven by reconnection and Pi1-2 waves are driven by current disruption, which would be observational evidence of the theory that current disruption occurs first and mid-tail reconnection occurs subsequently (e.g. Lui 1991; Pu et al. 1999). This is also consistent with the results of Rae et al. (2011) who performed a statistical study of the onset times of Pi1-2 and Pi2 waves at the location of substorm auroral brightening and found that Pi1-2 waves were observed consistently before the signatures in Pi2 waves.

## Conclusion

In this paper, we probe the relationship between three distinct ULF wave period bands and the initiation of a small substorm expansion phase onset using the AWESOME algorithm (Murphy et al. 2009a). We determine the relative timing between sudden increases in amplitude, or onsets, of different ULF wave bands at multiple ground-based magnetometer stations, and discuss how two separate theories of substorm onset dynamics can explain the wave characteristics observed.

We find that the onset of long-period Pi1/short-period Pi2 (Pi1-2) ULF waves are the first ULF wave signature observed at substorm onset followed by Pi2s and subsequently by Pi1s. The onset of the Pi1-2 waves is co-located in time and space with the formation of small-scale auroral beads. This is in contrast to the later arrival of Pi2 waves that occur in two distinct epicentres, one at auroral latitudes and one at lower latitudes. The onset of Pi1 waves occurs following both Pi1-2 and Pi2 onsets and is confined to latitudes near substorm onset. To date, there are no realistic travel times calculated for substorm onset, but the most realistic of any travel times is by Chi et al. (2009) using cold plasma theory using the onset time of Pi2 waves in the ionosphere as the marker for “Tamao travel times”. Indeed, Ferdouis and Raeder (2016) stated that the real MHD travel paths are more complicated than the Tamao travel path, but that there is good qualitative agreement between their studies and the predicted Tamao travel times.

We conclude that the differing onset times and spatial expansion of the Pi1, Pi1-2 and Pi2 waves are the result of the physical process which drives each wave in the ionosphere. Pi1-2 waves, being observed at onset and collocated with auroral beads, are the result of the initiation of a plasma instability in the inner magnetosphere which subsequently leads to the development of the substorm expansion phase. Pi2 waves, observed following Pi1-2 waves during the expansion phase, are the result of the development of the SCW and dipolarisation of the tail. Finally, Pi1 waves, occurring following both Pi1-2 and Pi2 waves during the expansion phase and localised to higher latitudes, are the result of filamentary currents and wave aurora developing during expansion phase as the aurora expands poleward and toward the dawn and dusk. These results demonstrate how careful analysis of ULF waves surrounding substorm onset can provide vital information on the physical processes occurring and time history of these processes through substorm onset.
